# Examining the role of elective pelvic radiotherapy in patients Diagnosed with high- and very High-Risk Non-Metastatic prostate cancer

**DOI:** 10.1016/j.ctro.2025.100960

**Published:** 2025-04-11

**Authors:** István Nahaji, Zsuzsa S. Kocsis, Andrea Kovács, Levente Varga, László Gesztesi, Kliton Jorgo, Zoltán Takácsi-Nagy, Csaba Polgár, Péter Ágoston

**Affiliations:** a National Institute of Oncology, Centre of Radiotherapy, Budapest, Hungary; b National Tumor Biology Laboratory, National Institute of Oncology, Budapest, Hungary; c Semmelweis University, Department of Oncology, Budapest, Hungary

## Abstract

**Introduction:**

In the treatment of node-negative, non-metastatic high-risk (HR) and very high-risk (VHR) prostate cancer, the necessity of elective pelvic irradiation is controversial. According to our in-house treatment protocol − elective pelvic irradiation is generally omitted for HR and VHR patients over the age of 70 or those in poor general health due to its toxicity.

**Objective:**

To retrospectively examine the outcome for HR and VHR prostate cancer patients treated with elective whole pelvic radiotherapy (WPRT) versus prostate-only radiotherapy (PORT).

**Materials and Methods:**

The study included 434 patients treated with definitive radiotherapy, 203 patients received PORT (HR: 127, VHR: 76) and 231 WPRT (HR: 113, VHR: 118) with a boost to the prostate. Patients also received 2–3 years of androgen deprivation. Patients’ average age who received PORT vs. WPRT was 73.9 ± 4.3 years vs. 66.4 ± 5.4 years respectively. An inverse propensity score weighting method was utilized to create homogeneous WPRT and PORT treatment groups that are balanced for T stage, PSA, and Gleason score, but not for age. The survival outcomes for HR and VHR subgroups were examined depending on whether they received WPRT or PORT. Biochemical- (BRFS), local- (LRFS) and regional relapse-free survival (RRFS), distant metastasis-free- (DMFS), disease-free- (DFS), failure-free- (FFS), and overall survival (OS) were compared using the Kaplan −Meier method and Cox regression analysis.

**Results:**

The median follow-up time was 76 months (3–134 months). In the VHR subgroup five-year outcomes showed a significant advantage for patients receiving WPRT vs. PORT in BRFS (82.2 % vs. 73 %; p = 0.028), in DMFS (87.5 % vs. 73.6 %; p = 0.025), in DFS (86.1 % vs. 70.5 %; p = 0.012), and in FFS (82.3 % vs. 68.9 %; p = 0.005), respectively. The OS (92.8 % vs. 81.8 %; p = 0.056) showed a trend favoring the WPRT group. There was no significant difference between WPRT vs. PORT in LRFS (95.8 % vs. 96.4 %; p = 0.763) and RRFS (95.8 % vs. 89.9 %; p = 0.099). On the contrary, in the HR group, no significant survival differences were observed between WPRT vs. PORT groups: BRFS 93.0 % vs. 93.3 % (p = 0.978), LRFS 99.0 % vs. 100 % (p = 0.120), RRFS 98.2 % vs. 95.1 % (p = 0.813), DMFS 93.5 % vs. 95.5 % (p = 0.793), DFS 91.7 % vs. 92.9 % (p = 0.691), FFS 89.5 % vs. 90.9 % (p = 0.853), OS 91.0 % vs. 87.7 % (p = 0.407).

**Conclusion:**

Based on our retrospective data elective pelvic irradiation can be omitted in HR patients, especially over the age of 70. For VHR patients, elective pelvic irradiation should be considered even for the subgroup of elderly patients.

## Highlights

• Pelvic radiotherapy in node-negative prostate cancer remains controversial.
• Pelvic radiotherapy improves survival outcomes in very high-risk prostate cancer.
• No clear benefit of pelvic radiotherapy for high-risk prostate cancer patients.
• Treatment should be personalized based on individual risk and health condition.

## Introduction

External beam radiotherapy, combined with androgen deprivation therapy (ADT), is one of the recommended primary treatment approaches in non-metastatic, high-risk (HR) or very high-risk (VHR) prostate cancer. However, the benefits of prophylactic radiotherapy of the pelvic lymph nodes for these patients without clinical regional pelvic lymph node involvement (cN0) have long been a topic of debate in the literature. Pelvic radiotherapy aims to eliminate suspected lymph node micrometastases, improving regional control and potentially improving survival outcomes[1]. Despite the long-term clinical results of randomized trials investigating this issue, the question remains unresolved[2,3,4]. Irradiation of the pelvic lymph node region can increase the rate of gastrointestinal and urinary side effects of the treatment[5,6], and even hematological toxicity can be caused by the irradiation of a larger volume of the bone marrow[7], therefore it would be important to select patients with HR and VHR prostate cancer who benefit from radiation treatment of the pelvic region. The current definitions of HR and VHR prostate cancer encompass patients with varying prognoses, necessitating distinct treatments in some cases. Defining VHR appropriately and identifying such patients is pivotal, as they may benefit from elective pelvic irradiation added to prostate- only radiotherapy[8]. According to our in house radiation therapy protocol, HR and VHR patients undergo WPRT, with a boost to the prostate and vesicles, except for patients over 70 years of age or patients with significant co-morbidities. In the letter cases, pelvic radiotherapy is usually omitted based on the decision of the treating physician. This provided the basis for examining the effect of pelvic radiotherapy in two groups of patients with prostate cancer different in age, but homogeneous otherwise regards to prognostic factors. In our current retrospective study, we are seeking the population of patients with HR or VHR prostate cancer in whom elective whole pelvic radiotherapy (WPRT) could improve relapse-free survival compared to those who underwent prostate-only radiotherapy (PORT).

Patients and Methods.

In our study, we conducted a retrospective analysis of consecutively treated patients with clinically localized or locally advanced, non-metastatic HR or VHR prostate cancer who received definitive radiotherapy combined with 2–3 years of hormone therapy between 2010 and 2016, according to our protocol. These patients had not previously undergone pelvic radiotherapy. HR patients were defined according to the risk stratification by D’amico et al., with clinical T stage ≥ T2c, Gleason score (GSC) ≥ 8, or prostate-specific antigen (PSA) ≥ 20 ng/ml [9]. Our institute defined VHR patients based on the risk stratification outlined by Spahn et al. and Walz et al., requiring a minimum of 2 high-risk criteria to be present[10,11]. Magnetic resonance imaging (MRI) of the pelvis or contrast-enhanced computer tomography (CT) scan of abdomen and pelvis, and bone scan were required for staging. According to risk classification we defined HR (n = 235) and VHR (n = 193) groups.

During PORT treatment, we administered 78 Gy in 2 Gy fractions (78/2 Gy), five times a week, with CT-planned photon irradiation, using intensity-modulated- and image-guided radiation therapy (IMRT/IGRT) techniques to the prostate and 60 Gy to the seminal vesicles. In some cases, external beam radiotherapy (EBRT) was combined with high dose-rate brachytherapy boost (HDR-AL)[12], where the boost of 2x10 Gy was given after 50/2 Gy fractionatied external beam therapy to the prostate and the seminal vesicles, or a boost of 1x10 Gy was given after 60/2 Gy fractionated photon irradiation to the prostate and seminal vesicles.

During WPRT treatment, we also performed CT-based treatment planning and used IMRT/IGRT techniques. We delivered 44/2 Gy fractionated photon irradiation to the pelvic region, followed by an additional 16/2 Gy fractionated photon irradiation to the prostate and seminal vesicles, and finally 18/2 Gy boost to the prostate gland reaching 78/2 Gy to the prostate. In certain cases, we administered 50/2 Gy fractionated photon irradiation to the prostate and seminal vesicle areas (of which 44/2 Gy was delivered to the pelvic region) with a 2x10 Gy HDR-AL boost to the prostate gland, or 60/2 Gy fractionated photon irradiation to the prostate and the vesicles with a 1x10 Gy HDR-AL boost to the prostate. In later years following evidence coming from randomized data on moderately hypofractionated radiotherapy[13], we introduced a simultaneous integrated boost (SIB) technique. where we applied 50.4/1.8 Gy fractionated photon irradiation to the pelvic region, 58.8/2.1 Gy to the seminal vesicles and 70/2.5 Gy to the prostate [14,15].

In our Radiotherapy Centre, according to our protocol, WPRT could be omitted for patients aged 70 + or those in poor health, at the physician's discretion, even if they were in the HR or VHR categories. However, for VHR patients in good health, WPRT could still be considered after 70 based on individual assessment Table 1.

**Table 1:** Characteristics of the original study population comparing patients receiving prostate-only and whole pelvic radiotherapy using the Chi-square test both in HR and VHR categories.

		original study population					
		HR (n = 240)			VHR (n = 194)		
		PORT (n = 127)	WPRT (n = 113)	p	PORT (n = 76)	WPRT (n = 118)	p
Mean age (± SD)		73.6 ± 4.7	66.8 ± 5.1	<0.0001	74.5 ± 3.7	66.2 ± 5.7	<0.0001
Mean iPSA (± SD)		17.8 ±14.0	30.4 ± 35.1	0.033	45.9 ± 39.5	50.8 ± 52.7	0.690
GSC	4–7 (%)	67.0	67.3	0.957	17.1	22.0	0.403
	8–10 (%)	33.0	32.7		82.9	78.0	
T	1 (%)	25.2	15.0	0.033	19.7	2.5	0.0005
	2 (%)	44.9	61.1		21.1	28.8	
	3 (%)	29.9	23.9		57.9	63.6	
	4 (%)	0	0		1.3	5.1	

HR: high-risk; VHR: very high-risk; PORT: prostate-only radiotherapy; WPRT: whole pelvis radiotherapy; iPSA: initial PSA; GSC: Gleason score; T: T stage.

As part of a statistical analysis, we utilized an inverse propensity score weighting (IPW) method to create homogeneous treatment groups regarding T stage, iPSA, GSC parameters, in order to compare the two treatments [16,17]. With this method, we constructed a pseudo-population from the real data. In the stabilized average treatment effect (ATE) weighted pseudo-population age differences between PORT and WPRT patients persisted, with PORT patients being significantly older than WPRT patients in both risk groups. Otherwise, there were no significant differences in iPSA levels, GSC, or T-stage between patients receiving PORT or WPRT in either the HR or VHR groups (see Table 2).

**Table 2:** Characteristics of the stabilised average treatment effect (ATE) weighted pseudo-population comparing receiving prostate-only and whole pelvic radiotherapy of patients using the Chi-square test both in HR and VHR categories.

stabilised ATE weighted pseudo-population							
		HR (n = 235)			VHR (n = 193)		
		PORT (n = 122)	WPRT (n = 113)	p	PORT (n = 77)	WPRT (n = 116)	p
Mean age ± SD		73.8 ± 4.6	66.7 ± 4.7	<0.0001	74.2 ± 4.3	66.4 ± 5.6	<0.0001
Mean iPSA ± SD		21.1 ± 19.9	23.4 ± 27.1	0.453	50.2 ± 42.5	48.0 ± 48.3	0.754
GSC	4–7 (%)	67.5	67.0	0.919	18.1	20.3	0.744
	8–10 (%)	32.5	33.0		81.9	79.7	
T	1 (%)	22.3	20.4	0.929	9.1	8.3	0.888
	2 (%)	48.9	51.2		26.7	26.5	
	3 (%)	28.8	28.4		58.5	61.5	
	4 (%)	0	0		5.7	3.7	

ATE: average treatment effect; HR: high-risk; VHR: very high-risk; PORT: prostate-only radiotherapy; WPRT: whole pelvis radiotherapy; iPSA: initial PSA; GSC: Gleason score; T: T stage.

The results were analyzed for multiple survival metrics, with percentages and standard error margins (SEM) reported, alongside statistical significance assessed through p-values (see Table 3). We examined the biochemical- (BRFS, from the end of the treatment to more than 2 ng/mL PSA increase above nadir), local- (LRFS, from the end of the treatment to local relapse), regional relapse-free- (RRFS, from the end of the treatment to local or regional relapse, which comes first), distant metastasis-free (DMFS, from the end of the treatment to distant metastasis), disease-free survival (DFS, from the end of the treatment to local, regional relapse or distant metastasis), failure-free survival (FFS, from the end of the treatment to biochemical, local, regional relapse or distant metastasis), overall survival (OS, from the end of the treatment to death) of patients who underwent PORT and WPRT treatments. We considered death as a censored event in BRFS, LRFS, RRFS, DMFS, DFS, FFS. The aim was to determine whether there is a difference in the survival parameters between WPRT and PORT treatment in patients with HR, and VHR prostate cancer who were matched based on inverse propensity score matching method.

**Table 3:** 5-year survival rates for HR and VHR patients treated (according to risk stratification by Spahn and Walz[10,11]) with prostate-only and whole pelvic radiotherapy.

	**Risk stratification according to Spahn et al. & Walz et. al.** [12,13]									
5-year results	HR (n = 235)					VHR (n = 193)				
	PORT	SEM	WPRT	SEM	p	PORT	SEM	WPRT	SEM	p
**BRFS**	93.3	2.5	93.0	2.6	0.978	73.0	5.6	82.2	3.8	0.028
**LRFS**	100		99.0	1.0	0.120	96.4	2.6	95.8	2.1	0.763
**RRFS**	95.1	2.2	98.2	1.3	0.813	89.9	4.0	95.8	2.1	0.099
**DMFS**	95.5	2.0	93.5	2.6	0.793	73.6	5.7	87.5	3.4	0.025
**DFS**	92.9	2.4	91.7	2.8	0.691	70.5	5.9	86.1	3.5	0.012
**FFS**	90.9	2.8	89.5	3.2	0.853	68.9	5.8	82.3	3.8	0.005
**OS**	87.7	3.2	91.0	2.9	0.407	81.8	5.4	92.8	2.7	0.056

Biochemical relapse − (BRFS), local relapse- (LRFS), regional relapse- (RRFS), distant metastasis-free- (DMFS), disease-free- (DFS), failure-free- and overall survival (OS). High-risk (HR), very high-risk (VHR), prostate-only radiotherapy (PORT), standard error margins (SEM), whole-pelvic radiotherapy (WPRT).

Chi-squared and Mann-Whitney u-test was used to compare the baseline characteristics of the treatment groups. Propensity scores were balanced for T status, PSA and Gleason scores, and converted to ATE stabilized weights[16,17]. This IPW was used in the Chi-squared and Mann-Whitney u-test of the pseudo-population and Kaplan-Meier statistics. Statsoft Statistica version 12 and GraphPad Prism was used for data analysis and representation. Standard error of means (SEM) calculated by Statsoft Statistica from the weighted Kaplan-Meier curves are reported. The numbers at risk are calculated with propensity score weighting of life tables by Statistica 12.

## Results

A total of 434 patients were included in the study. For patients who underwent WPRT, the median follow-up was 77 months (ranging from 3 to 134, SEM = 2.9), whereas for those who underwent PORT, the median follow-up was 72 months (ranging from 6 to 130, SEM = 3.5). Among patients who received PORT treatment, the average age was 73.9 ± 4.3 years, the average initial (pre-treatment) prostate-specific antigen (iPSA) value was 28.3 ± 29.8 ng/ml. In 48.3 % (n = 98) they had GS 4–7, and in 51.7 % (n = 105) had GS 8–10, 59.1 % (n = 120) of them were classified with T1-2, and 40.9 % (n = 83) of them with T3-4 tumours.

For patients who underwent WPRT treatment, the average age was 66.4 ± 5.4 years, the average iPSA value was 40.8 ± 46.9 ng/ml. 44.2 % of them (n = 102) had GS 4 to 7, and 55.8 % of them (n = 129) had GS 8–10, 53.2 % of them (n = 123) were classified with T1-2, and 46.8 % of them (n = 108) with T3-4 tumours.

The HR and the VHR groups were further subdivided based on the type of radiation therapy: PORT or WPRT. HR group: PORT patients’ mean age was 73.6 ± 4.7 years, while WPRT patients were younger with a mean age of 66.8 ± 5.1, the difference in age was significant (p < 0.0001, Chi-square test). VHR group: PORT patients’ mean age was 74.5 years ± 3.7, while WPRT patients were younger with a mean age of 66.2 ± 5.7 years. This difference was also statistically significant (p < 0.0001). In the HR group PORT patients had a mean iPSA of 17.8 ± 14.0 ng/ml, while WPRT patients had a higher mean iPSA of 30.4 ± 35.1 ng/ml, with a statistically significant difference between them (p = 0.033). In VHR group the mean iPSA for PORT patients was 45.9 ± 39.5 ng/ml, and for WPRT patients it was 50.8 ± 52.7 ng/ml. This difference was not statistically significant (p = 0.690). The GSC distribution between PORT and WPRT patients in both the HR and VHR groups showed no statistically significant differences (HR p = 0.957, VHR p = 0.403). In both the HR and VHR groups, patients receiving WPRT tended to have higher T-stages (HR p = 0.033, VHR p = 0.0005) (see Table 1).

After inverse propensity score weighting, the ATE weighted pseudo-population was more balanced (Table 2). Neither the iPSA, Gleason-score nor the T stage differed significantly.

We performed the survival analysis on the pseudo-populations. For BRFS no significant differences were observed in the HR group, where PORT achieved 93.3 % (SEM ± 2.5) and WPRT 93.0 % (SEM ± 2.6; p = 0.978). However, in the VHR group, BRFS was significantly higher with WPRT (82.2 %, SEM ± 3.8) compared to PORT (73.0 %, SEM ± 5.6; p = 0.028), suggesting improved efficacy with WPRT for this higher-risk subgroup. In terms of LRFS the HR group showed a 100 % LRFS rate with PORT, while WPRT achieved 99.0 % (SEM ± 1.0; p = 0.120), indicating no significant difference. Similarly, for the VHR group, LRFS rates were comparable between PORT (96.4 %, SEM ± 2.6) and WPRT (95.8 %, SEM ± 2.1; p = 0.763), with no statistical significance detected. Analysis of RRFS revealed no significant difference in the HR group, where PORT reached 95.1 % (SEM ± 2.2) and WPRT achieved 98.2 % (SEM ± 1.3; p = 0.813). In the VHR group, RRFS was slightly higher with WPRT (95.8 %, SEM ± 2.1) compared to PORT (89.9 %, SEM ± 4.0), with a trend towards significance (p = 0.099). For DMFS the HR group again showed no significant difference, with PORT achieving 95.5 % (SEM ± 2.0) and WPRT 93.5 % (SEM ± 2.6; p = 0.793). In contrast, the VHR group demonstrated significantly improved DMFS with WPRT (87.5 %, SEM ± 3.4) compared to PORT (73.6 %, SEM ± 5.7; p = 0.025). DFS followed a similar pattern. In the HR group, DFS rates were 92.9 % (SEM ± 2.4) for PORT and 91.7 % (SEM ± 2.8) for WPRT, with no significant difference (p = 0.691). In the VHR group, WPRT showed superior DFS at 86.1 % (SEM ± 3.5) compared to 70.5 % (SEM ± 5.9) for PORT, with statistical significance (p = 0.012). The comparison of FFS in the HR group yielded similar results for both treatments, with PORT achieving 90.9 % (SEM ± 2.8) and WPRT 89.5 % (SEM ± 3.2; p = 0.853). In the VHR group, FFS was significantly higher with WPRT (82.3 %, SEM ± 3.8) than with PORT (68.9 %, SEM ± 5.8; p = 0.005). For OS the HR group showed no significant differences, with PORT achieving 87.7 % (SEM ± 3.2) and WPRT 91.0 % (SEM ± 2.9; p = 0.407). In the VHR group, OS was higher with WPRT (92.8 %, SEM ± 2.7) compared to PORT (81.8 %, SEM ± 5.4), with a trend (p = 0.056).

**Fig. 1:**
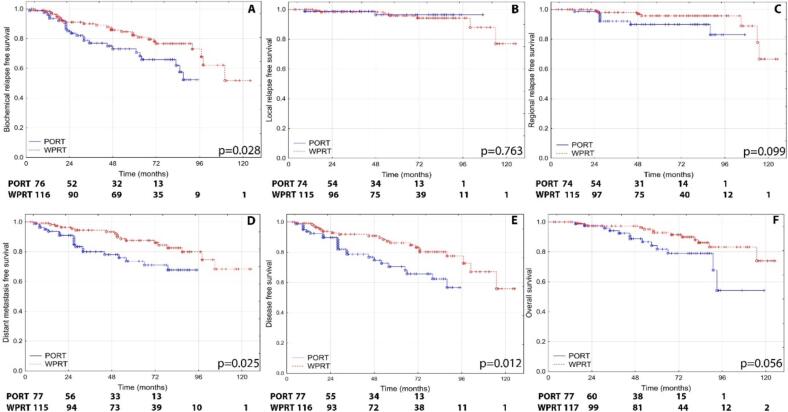
Kaplan-Meier curves and numbers at risk of (A) biochemical relapse-free survival, (B) local relapse-free survival, (C) regional relapse-free survival, (D) distant metastasis-free survival, (E) disease-free survival and (F) overall survival of very high risk patients, who received prostate- only (PORT) or whole pelvic irradiation (WPRT).

**Fig. 2:**
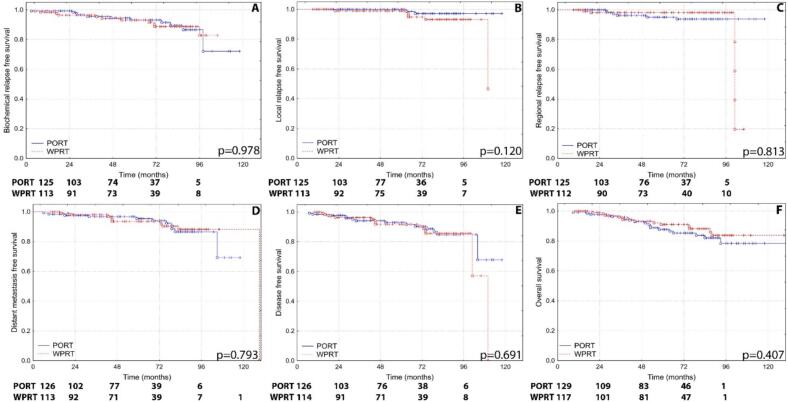
Kaplan-Meier curves and numbers at risk of (A) biochemical relapse free survival, (B) local relapse free survival, (C) regional relapse free survival, (D) distant metastasis free survival, (E) disease free survival and (F) overall survival of high risk patients, who received prostate-only (PORT) or whole pelvic irradiation (WPRT).

In summary, while both PORT and WPRT demonstrated comparable efficacy in the HR group across most survival metrics, WPRT provided significant improvements in BRFS, DMFS, DFS, and FFS in the VHR group, with additional non-significant benefits for RRFS and OS (see Fig. 1, Fig. 2).

*The numbers at risk are calculated with propensity score weighting of life tables by Statistica 12.*

*The numbers at risk are calculated with propensity score weighting of life tables by Statistica 12.*

## Discussion

A prospective randomized phase III trial (POP-RT), published in 2021, showed significant differences in BRFS, DMFS and DFS in patients suffering from HR and VHR prostate cancer receiving PORT only or WPRT with a boost to the prostate [4]. In this study, the parameters of HR and VHR patients were not analysed separately, but a subgroup analysis was performed, and it was concluded that younger patients had a higher benefit of WPRT in BRFS and DFS.

The present study aimed to evaluate the impact of two radiotherapy approaches—PORT and WPRT—on patients with HR and VHR prostate cancer, based on their survival outcomes. By employing inverse propensity score weighting for balancing patient characteristics and comparing BRFS, DMFS, DFS, FFS, and OS, we sought to identify differences between these treatment modalities.

In original study population both the HR and VHR groups, patients who underwent PORT were significantly older and had less advanced disease compared to those receiving WPRT. This suggests that WPRT may be more frequently administered to younger patients − in concordance with our in-house protocol − with more aggressive disease, likely reflecting physician preferences for offering more extensive treatment to patients expected to tolerate it better.

The survival analysis applied in this study was executed in stabilized ATE-weighted pseudo-population and yielded distinct results for the HR and VHR groups. In the HR group, no significant differences were observed between PORT and WPRT for any of the survival outcomes, including BRFS, DMFS, DFS, and OS. These results indicate that both treatment modalities may be equally effective for patients with HR prostate cancer, suggesting that WPRT may not confer additional survival benefits over PORT in this cohort. This finding aligns[18,19] with previous studies that have reported comparable outcomes between PORT and WPRT in certain HR prostate cancer populations, and raises the question of whether the added toxicity of WPRT is justified in these patients.

In contrast, among VHR patients, WPRT demonstrated a clear advantage over PORT in terms of several key outcomes. Specifically, WPRT was associated with significantly better BRFS, DMFS, DFS, and FFS compared to PORT, with a trend toward improved OS as well. The survival advantage in BRFS (p = 0.028) and DFS (p = 0.012) suggests that WPRT more effectively reduces biochemical recurrence and delays disease progression. The significant improvement in DMFS (p = 0.025) further highlights the role of WPRT in reducing the risk of distant metastasis, which is a critical concern in VHR prostate cancer. These findings support the use of WPRT in VHR patients, as it appears to improve both locoregional control and systemic disease outcomes.

The lack of significant differences in LRFS between PORT and WPRT in both risk groups is notable, as local control was near perfect in both treatment arms. This suggests that both PORT and WPRT are effective in controlling local disease, regardless of risk category. However, the advantage of WPRT in VHR patients seems to stem from its ability to control regional and distant disease, which likely contributes to the improved overall survival trends observed in this group.

The use of IPW stabilized weighting to create a pseudo-population for further analysis allowed us to adjust for baseline differences between treatment groups, confirming that the survival advantages of WPRT in the VHR group were not solely due to the selection of younger, healthier patients with more aggressive disease.

While WPRT was associated with better outcomes in VHR patients, the treatment decisions should also consider potential toxicity. Although our study did not specifically evaluate treatment-related adverse effects, previous studies have suggested that WPRT may increase gastrointestinal and genitourinary toxicity compared to PORT. It is more so if the patient is older or he has bowel disease or other serious comorbidities deteriorating the vasculature of the bowel and the bladder[20,21,22]. Also secondary cancer should be considered, as higher treated volume potentially can cause higher rate of secondary cancer due to radiation therapy[23,24]. This potential trade-off between survival benefits and treatment-related morbidity should be carefully considered, particularly for older patients or those with comorbidities.

It is important to note that at the time of our treatments of the study population, functional imaging (PSMA PET-CT) was not yet available in Hungary, therefore in clinical staging we relied on classical imaging (pelvic MRI, thoracal CT, bone scan). In our current experience, PSMA PET-CT often indicates a pathological lymph node in the pelvis without its malignancy being detected by conventional imaging. Nevertheless, only few regional relapses developed in both study groups.

The benefit observed in the VHR WPRT group might be because even pathological lymph nodes not visible on imaging were treated with ADT + RT, whereas in the HR group the benefit is lost because, given the less aggressive nature of the disease, micrometastatic disease can be controlled with ADT alone. Although we also know that hormone therapy never controls prostate cancer perfectly and the median follow-up time in our study was four years longer than the hormone period, which means that we probably measured the differences in outcome made mostly by the radiotherapy[25,26].

## Conclusion

This retrospective analysis suggests that WPRT may offer significant survival benefits over PORT in patients with VHR prostate cancer, particularly in terms of BRFS, DMFS, DFS, and FFS. However, for HR patients, no significant differences in survival outcomes were observed between the two treatment modalities. These findings highlight the importance of tailoring radiotherapy based on individual risk stratification, with WPRT being more beneficial in patients with localized, or locally advanced prostate cancer and the highest risk of disease progression.

In the future, we are planning to start a prospective randomized study using the results of this retrospective analysis showing better survival with WPRT in VHR patients and also the results of the first randomized trial (POP-RT) in this issue showing a clear benefit in outcome with WPRT in HR and VHR risk patients, to further investigate the best patient selection for elective pelvic radiotherapy.

## Declaration of Competing Interest

The authors declare that they have no known competing financial interests or personal relationships that could have appeared to influence the work reported in this paper.
